# Cell-specific RNA profiling reveals host genes expressed in Arabidopsis cells haustoriated by downy mildew

**DOI:** 10.1093/plphys/kiad326

**Published:** 2023-06-12

**Authors:** Shuta Asai, Volkan Cevik, Jonathan D G Jones, Ken Shirasu

**Affiliations:** Center for Sustainable Resource Science, RIKEN, 1-7-22 Suehiro-cho, Tsurumi, Yokohama, Kanagawa 230-0045, Japan; Department of Life Sciences, The Milner Centre for Evolution, University of Bath, Bath BA2 7AY, UK; The Sainsbury Laboratory, Norwich Research Park, Norwich NR4 7UH, UK; Center for Sustainable Resource Science, RIKEN, 1-7-22 Suehiro-cho, Tsurumi, Yokohama, Kanagawa 230-0045, Japan

## Abstract

The downy mildew oomycete *Hyaloperonospora arabidopsidis*, an obligate filamentous pathogen, infects Arabidopsis (*Arabidopsis thaliana*) by forming structures called haustoria inside host cells. Previous transcriptome analyses have revealed that host genes are specifically induced during infection; however, RNA profiling from whole-infected tissues may fail to capture key transcriptional events occurring exclusively in haustoriated host cells, where the pathogen injects virulence effectors to modulate host immunity. To determine interactions between Arabidopsis and *H. arabidopsidis* at the cellular level, we devised a translating ribosome affinity purification system using 2 high-affinity binding proteins, colicin E9 and Im9 (immunity protein of colicin E9), applicable to pathogen-responsive promoters, thus enabling haustoriated cell-specific RNA profiling. Among the host genes specifically expressed in *H. arabidopsidis*–haustoriated cells, we found genes that promote either susceptibility or resistance to the pathogen, providing insights into the Arabidopsis–downy mildew interaction. We propose that our protocol for profiling cell-specific transcripts will apply to several stimulus-specific contexts and other plant–pathogen interactions.

## Teaser

A translating ribosome affinity purification system is used to analyze gene expression in pathogen-infected host cells and identify host genes whose expression is induced in infected cells.

## Introduction

*Hyaloperonospora arabidopsidis* causes downy mildew disease in the model plant Arabidopsis (*Arabidopsis thaliana*). *Hyaloperonospora arabidopsidis* is an obligate biotrophic oomycete that completes its life cycle without killing the host. Asexual *H. arabidopsidis* conidiospores germinate and form appressoria to penetrate leaf surfaces. Hyphae then grow intercellularly, producing numerous pyriform-shaped structures called haustoria in mesophyll cells (Coates and Beynon 2010). Haustoria impose invaginations on the plant cell, creating an interface between host and pathogen called an extra-haustorial matrix. This matrix is thought to be the site where the pathogen acquires nutrients from the plant and where pathogen-derived effectors are delivered into the host cell to suppress defense responses and promote susceptibility.

**Figure 1.**
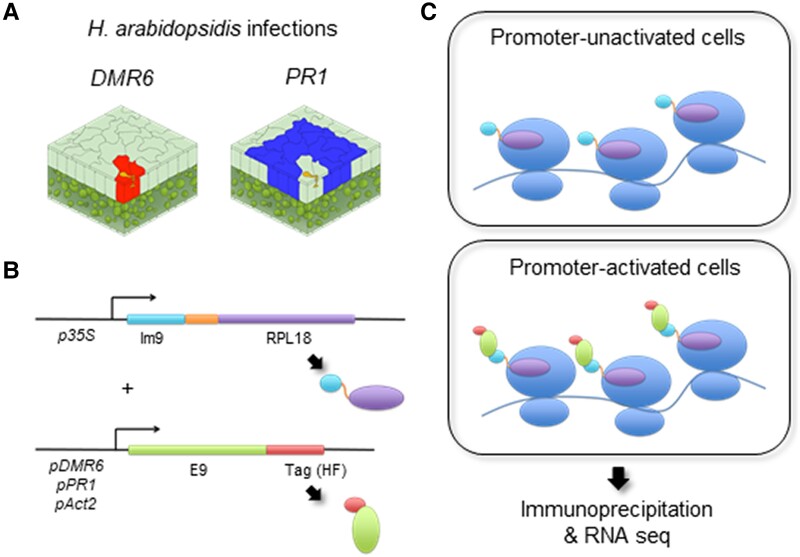
Schematic diagram of the TRAP system. **A)** Schematic view of cell-specific responses in the *H. arabidopsidis*–Arabidopsis interaction. *Hyaloperonospora arabidopsidis* extends hyphae to form haustoria inside host cells (yellow shapes). Red-shaded cells indicate cells in which the *DMR6* promoter (*pDMR6*) is activated, i.e. the haustoriated (infected) cells. Blue-shaded cells indicate cells in which the *PR1* promoter (*pPR1*) is activated, i.e. the nonhaustoriated adjacent (noninfected) cells. The images were adopted from Ghareeb et al. (2020). **B)** Schematic representation of 2 chimeric constructs; Im9-RPL18 fused to the *35S* promoter (*p35S*) and E9-HF controlled by *pDMR6*, *pPR1*, or the *Actin2* promoter (*pAct2*). HF, a tandem 6xHis and 3xFLAG epitope tag. **C)** Schematic diagram of ribosomal complexes in cells where the promoters fused to E9-HF are unactivated (upper panel) or activated (lower panel).

Host genes that promote susceptibility to pathogens are called susceptibility (*S*) genes (van Schie and Takken 2014). *S* genes are generally expressed in infected cells to accommodate pathogens. In the Arabidopsis–downy mildew interaction, for example, the *S* gene *DMR6* (*DOWNY MILDEW RESISTANT 6*) is predominantly induced in host cells containing haustoria (haustoriated cells; Fig. 1A, van Damme et al. 2008). *DMR6* encodes a salicylic acid (SA) 5-hydroxylase that inactivates SA, a phytohormone essential for plant immunity (Zhang et al. 2017). Consistently, *H. arabidopsidis* specifically suppresses SA-inducible *PR1* (*PATHOGENESIS-RELATED GENE1*) expression in haustoriated cells, whereas *PR1* is expressed in the surrounding cells (nonhaustoriated cells; Fig. 1A; Caillaud et al. 2013; Asai et al. 2014). Several *H. arabidopsidis* effectors are able to suppress the SA-signaling pathway (Caillaud et al. 2013; Asai et al. 2014; Wirthmueller et al. 2018); however, little is known about what events occur in the infected cells to modulate the local responses of Arabidopsis to *H. arabidopsidis.* Identifying these events requires cell-specific transcript analysis.

Translating ribosome affinity purification (TRAP) is a powerful method that enables cell-type-specific RNA profiling (Mustroph et al. 2009b; Heiman et al. 2014; Fröschel et al. 2021). In the traditional TRAP system, ribosome-associated mRNAs are immunopurified from specific cell populations that express an epitope-tagged ribosomal protein via developmentally regulated promoters (i.e. cell-type-specific promoters; Supplemental Fig. S1; Mustroph et al. 2009b). Recently, cell-type-specific RNA profiling unraveled responses for each root layer in root–microbe interactions (Fröschel et al. 2021). A limitation of the traditional TRAP methodology makes the procedure inapplicable to cells in which stress-responsive promoters are activated because the newly synthesized epitope-tagged ribosomes must replace preexisting ribosomes in the cells, i.e. a problem of ribosomal turnover, where half of the ribosomes are replaced every 3 to 4 d in Arabidopsis (Salih et al. 2020). To overcome this limitation, an affinity tag, but not a ribosomal protein, should be controlled by the specific promoter to capture ribosomes with corresponding tags under the control of their own or a constitutive promoter. Based on this concept, we established a TRAP system that relies on high-affinity colicin E9-Im9 (immunity protein of colicin E9)-based interactions (*K*_d_ = 9.3 × 10^−17^ M; Wallis et al. 1995). Our system allows the formation of tagged ribosomal complexes predominantly in cells where the *DMR6* promoter is activated, thereby enabling haustoriated cell-specific RNA profiling. Among the haustoriated cell-specific transcripts, we found genes involved in resistance and susceptibility to *H. arabidopsidis*, indicating that haustoriated cell-specific RNA profiling can provide insights into the interaction between Arabidopsis and the downy mildew pathogen.

## Results

### A TRAP system for cells with specific promoter activation

Although *DMR6* and *PR1* show distinct cellular expression patterns in Arabidopsis infected with *H. arabidopsidis* (Fig. 1A; van Damme et al. 2008; Caillaud et al. 2013; Asai et al. 2014), transcriptome analysis using whole tissues revealed no substantial difference in the expression patterns of these genes during infection (Supplemental Fig. S2; Asai et al. 2014). To elucidate the interaction between Arabidopsis and *H. arabidopsidis* at the cellular level, we designed a TRAP system using 2 high-affinity binding proteins: a bacterial toxin protein, E9, and its cognate immunity protein, Im9 (Wallis et al. 1995). This TRAP system consists of 2 chimeric transgenes: 1 gene encodes RPL18 (RIBOSOMAL PROTEIN L18) fused to Im9 driven by the *35S* promoter (*p35S*); the second gene is controlled by promoters of stress-responsive genes such as *DMR6* (*pDMR6*) or *PR1* (*pPR1*) and encodes E9 fused to a tandem 6xHis and 3xFLAG epitope tag (HF) used for purification (Fig. 1B). In cells where the corresponding promoters are active, the purification tag attaches to ribosomes when binding between E9 and Im9 occurs (Fig. 1C).

**Figure 2.**
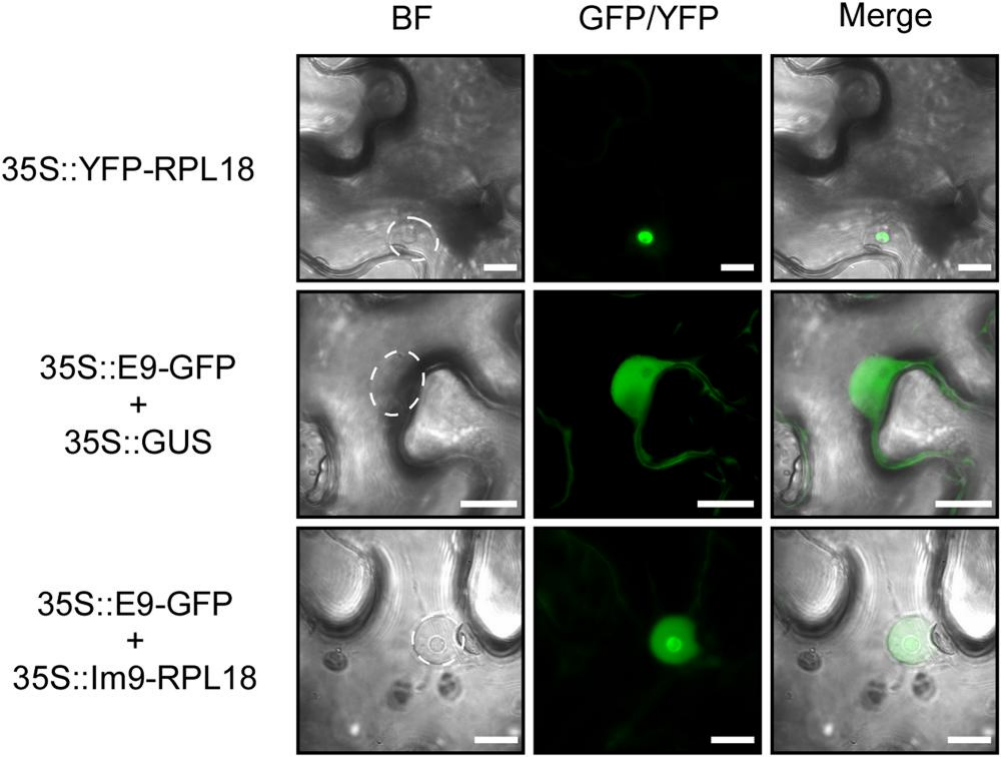
Formation of ribosomal complexes consisting of chimeric constructs coincident with E9 and Im9 binding. Subcellular localization of YFP-RPL18 and E9-GFP when coexpressed with GUS and Im9-RPL18. The indicated constructs were transiently expressed in *N. benthamiana* leaves. The left image is the bright-field (BF) image, the middle image is from the GFP/YFP channel, and the right image is the overlay of the BF image and GFP channel. Dashed white circles mark the locations of nuclei in the BF pictures. Scale bars, 10 *μ*m.

We confirmed whether tagged ribosomes are formed by the binding of E9 and Im9 using a *Nicotiana benthamiana* transient expression system. As expected, yellow fluorescent protein (YFP)-RPL18 accumulated in the nucleolus, where most ribosome biogenesis events take place (Fig. 2). E9-GFP (green fluorescent protein) localized to the cytoplasm and nucleus, excluding the nucleolus, when coexpressed with β-glucuronidase (GUS) as a control, whereas GFP fluorescence was observed in the nucleolus when E9-GFP was coexpressed with Im9-RPL18 (Fig. 2). These results indicated that ribosomal complexes consisting of chimeric constructs were formed upon the binding of E9 and Im9.

### Validating the cell-specific TRAP system with *H. arabidopsidis*–infected Arabidopsis

**Figure 3.**
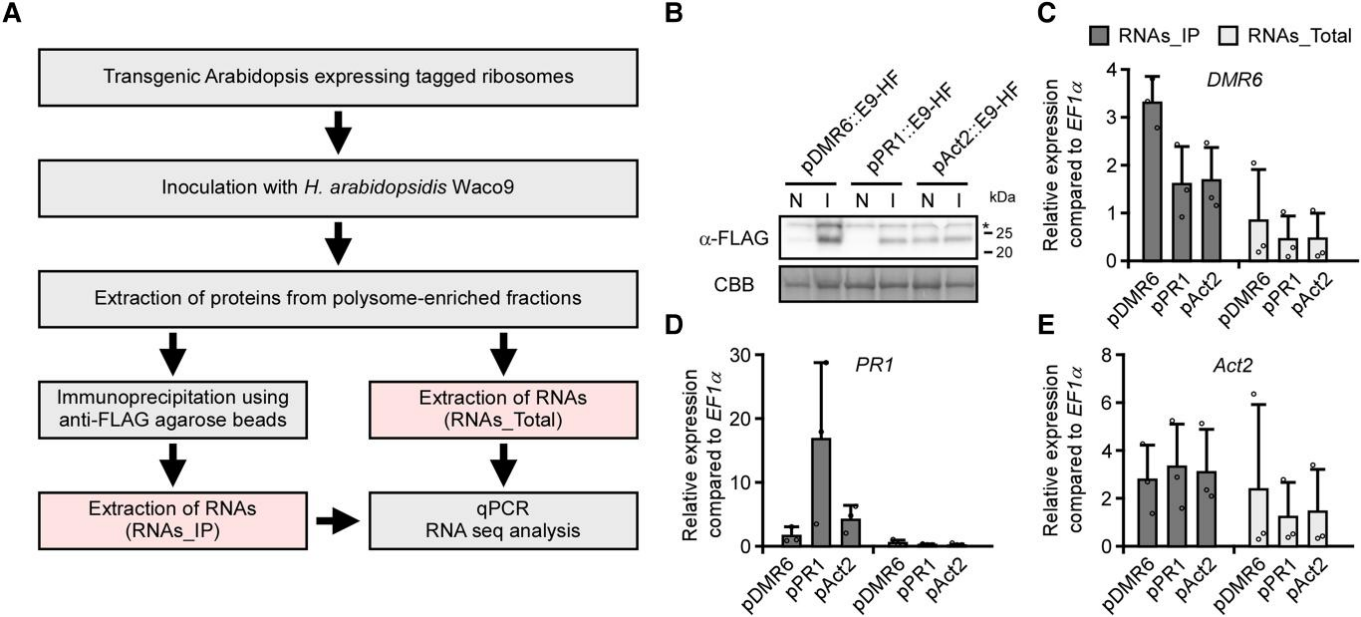
Validating the enrichment of specific cell-derived mRNAs during *H. arabidopsidis* infection by our TRAP system. **A)** Flow chart of the steps used to validate the cell-specific TRAP system. Protein accumulation **B)** and expression of *DMR6***C)**, *PR1***D)**, and *Act2***E)** in Arabidopsis Col-0 transgenic lines containing *pDMR6*::*E9-HF* (pDMR6), *pPR1*::*E9-HF* (pPR1) or *pAct2*::*E9-HF* (pAct2) and *p35S*::*Im9-RLP18*. **B)** Total proteins were prepared from 3-wk-old plants at 5 d after spraying water (N) or inoculation with *H. arabidopsidis* (I). An immunoblot analyzed using anti-FLAG (upper panel) antibodies. Protein loads were monitored by Coomassie brilliant blue (CBB) staining of bands corresponding to ribulose-1,5-bisphosphate carboxylase (Rubisco) large subunit (lower panel). **C to E)** The expression levels of *DMR6*, *PR1*, and *Act2* in the RNAs_IP and RNAs_Total samples were determined by RT-qPCR. Data are means ± Sds from 3 biological replicates.

We created Arabidopsis transformants containing 2 transgenes: Im9-RPL18 controlled by *p35S* (*p35S*::*Im9-RPL18*) and E9-HF driven by either *pDMR6* (*pDMR6*::*E9-HF*), *pPR1* (*pPR1*::*E9-HF*), or the *Actin2* promoter (*pAct2*::*E9-HF*) as a control (Fig. 1B). We hypothesized that E9-RPL18 would bind to Im9-HF in cells where both transgenes were expressed, thereby enabling conditional but efficient tagging of preexisting ribosomes in the cells of interest (Fig. 1C). An inoculum concentration of 1 × 10^4^ conidiospores mL^−1^ and 5 d post inoculation were selected as sufficient and nonsaturating infection conditions for the interaction between Arabidopsis Col-0 and *H. arabidopsidis* virulent isolate Waco9. After inoculating the transformants with *H. arabidopsidis* Waco9, proteins derived from fractions containing ribosomes and mRNAs (polysome-enriched fractions, see Materials and Methods) were extracted from infected tissues. The Im9-RPL18/E9-HF complexes were immunoprecipitated with anti-FLAG agarose beads, from which RNAs were extracted and referred to as RNAs_IP (Fig. 3A). We also extracted RNAs directly from the polysome-enriched fractions and designated those as RNAs_Total. To confirm whether E9-HF is properly controlled by *pDMR6* or *pPR1* in the transformants, immunoblots of protein samples after inoculation with *H. arabidopsidis* were probed with anti-FLAG antibodies. As expected, E9-HF was detected during *H. arabidopsidis* infection in transformants containing *pDMR6*::*E9-HF* or *pPR1*::*E9-HF*, whereas transformants containing *pAct2*::*E9-HF* constantly accumulated E9-HF (Fig. 3B). Importantly, reverse transcription-quantitative PCR (RT-qPCR) analysis confirmed that *DMR6* or *PR1* transcripts were enriched in the RNAs_IP samples derived from transformants containing *pDMR6*::*E9-HF* or *pPR1*::*E9-HF*, respectively, whereas the transcript levels of *Act2* were comparable among the RNAs_IP samples (Fig. 3, C to E). In the RNAs_Total samples, there was no difference in the transcript levels of either *DMR6*, *PR1*, or *Act2* (Fig. 3, C to E). These results indicated that our TRAP system successfully enriched specific cell-derived mRNAs during *H. arabidopsidis* infection.

### Identifying *DMR6*-coexpressed genes during *H. arabidopsidis* infection

**Figure 4.**
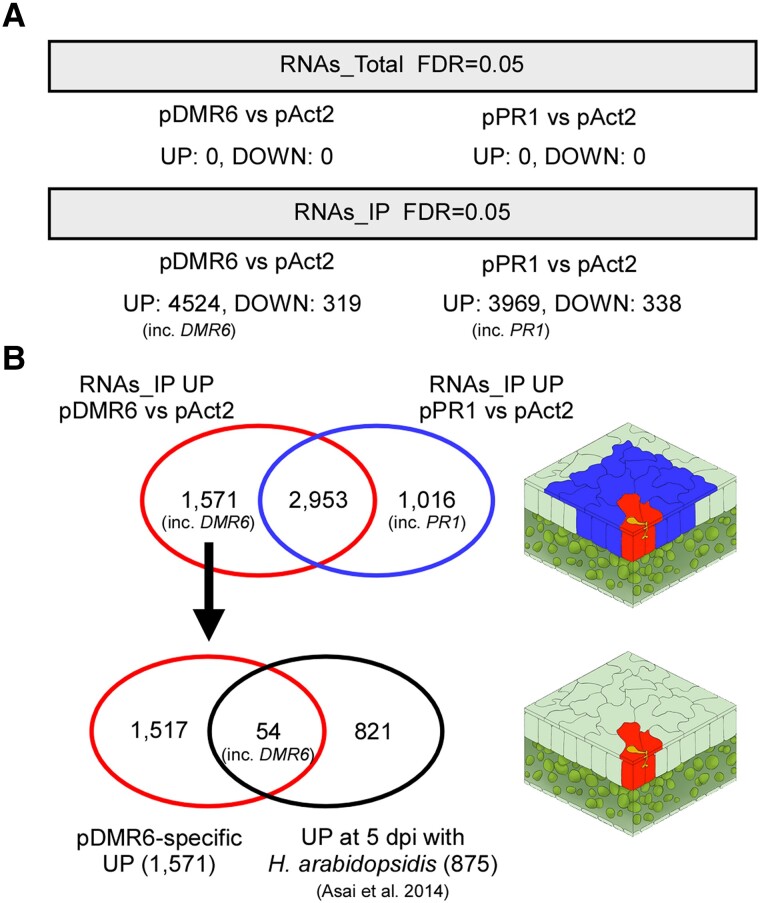
Selecting confident candidate *DMR6*-coexpressed genes. **A)** The number of genes significantly upregulated (UP) or downregulated (DOWN) among Arabidopsis Col-0 transgenic lines containing *pDMR6*::*E9-HF* (pDMR6), *pPR1*::*E9-HF* (pPR1), or *pAct2*::*E9-HF* (pAct2) and *p35S*::*Im9-RLP18*. **B)** Assessment of overlapping differentially expressed genes to select confident candidate *DMR6*-coexpressed genes. The comparison of upregulated genes between pDMR6 and pPR1 transformants in the RNAs_IP samples revealed 1,571 genes as *DMR6*-coexpressed candidate genes (pDMR6-specific UP). Comparing the 1,571 genes with 875 genes significantly upregulated at 5 d post inoculation (dpi) with *H. arabidopsidis* reported by Asai et al. (2014) revealed that 54 genes overlapped in the 2 conditions. The figures on the right indicate proposed expression sites: red-shaded cells, expression sites where *DMR6*-coexpressed genes are expressed; blue-shaded cells, expression sites where *PR1*-coexpressed genes are expressed.

To investigate cell-specific responses during *H. arabidopsidis* infection, the TRAP samples were subjected to RNA-seq analysis with 3 independent biological replicates (Supplemental Fig. S3 and Table S1). In the RNAs_Total samples, there were no differentially expressed genes in the *pDMR6*::*E9-HF* or the *pPR1*::*E9-HF* transformants compared with the *pAct2*::*E9-HF* control (Fig. 4A). By contrast, the RNAs_IP samples had genes with significant differences in expression levels (false discovery rate = 0.05). The *pDMR6*::*E9-HF* transformants had 4,524 upregulated genes and 319 downregulated genes; whereas the *pPR1*::*E9-HF* transformants had 3,969 upregulated genes and 338 downregulated genes compared with the *pAct2*::*E9-HF* control (Fig. 4A and Supplemental Table S2). Importantly, *DMR6* and *PR1* were among the upregulated genes of the *pDMR6*::*E9-HF* and *pPR1*::*E9-HF* transformants, respectively. To identify genes coexpressed with *DMR6* and specifically expressed in cells infected by *H. arabidopsidis* (haustoriated cells), we compared the upregulated genes in the *pDMR6*::*E9-HF* transformants to those in the *pPR1*::*E9-HF* transformants. The comparison revealed 1,571 candidate genes coexpressed with *DMR6* but not *PR1* (Fig. 4B and Supplemental Table S3). Candidate genes were further limited by a comparison with our previously reported list of genes whose expression was significantly upregulated during infection with *H. arabidopsidis* (Supplemental Table S3; Asai et al. 2014). In this analysis, we identified *DMR6* and 53 genes that were designated *DMR6*-coexpressed genes because they are very likely to be genes whose expression is induced in cells where *DMR6* is expressed when infected with *H. arabidopsidis* (Table 1). Among these 54 genes, gene ontology (GO) analysis revealed an overrepresentation of genes related to disease resistance (e.g. GO:0050832 and GO:0006952) and genes responsive to oxygen levels (e.g. GO:0001666 and GO:0070482) and chemicals (e.g. GO:0042221; Supplemental Fig. S4).

**Table 1.** The list and expression patterns of *DMR6* and 53 *DMR6*-coexpressed genes

		RNAs_IP			RNAs_Total		
AGI ID^a^	Name	pDMR6^b^	pPR1^b^	pAct2^b^	pDMR6^b^	pPR1^b^	pAct2^b^
AT1G02920	GSTF7, GST11	6,730.1	3,937.5	3,486.3	2,926.6	2,604.7	2,279.0
AT1G02930	GSTF6, GST1	10,346.6	5,948.0	6,299.9	4,218.3	3,778.1	3,295.7
AT1G05340	HCYSTM1	1,078.1	351.8	402.4	127.8	117.5	97.7
AT1G08310		9.1	2.2	0	0.9	2.0	2.9
AT1G08860	BON3	6.8	0	0	1.1	3.6	3.4
AT1G09080	BIP3, HSP70-13	4.9	1.9	0	4.8	8.1	5.7
AT1G09932		2,023.8	822.4	938.7	667.8	589.9	560.5
AT1G14870	PCR2	1,678.5	1,121.3	855.4	390.2	358.5	283.5
AT1G15010		416.7	159.5	79.1	45.7	53.6	51.8
AT1G21400	E1A1	128.6	79.5	14.1	91.5	65.8	60.9
AT1G34420		31.7	1.1	0	14.5	10.8	12.1
AT1G56060	HCYSTM3	271.1	210.3	96.8	29.0	48.4	57.4
AT1G65240	A39	17.3	5.3	0	1.7	3.0	1.1
AT1G65845		448.3	345.2	218.4	187.5	171.6	153.0
AT1G70170	MMP	22.4	3.4	0	7.2	7.7	6.9
AT1G73260	KTI1, KTI4	1,120.0	626.5	352.9	319.5	297.2	220.9
AT1G73810		18.9	16.7	1.9	24.1	20.4	20.2
AT1G78190	TRM112A	7.6	1.4	0	1.1	0.5	3.9
AT2G27389		104.3	13.8	6.5	10.0	15.6	11.7
AT2G28710		10.2	3.0	0	2.3	1.0	0.3
AT2G38870		1,134.0	753.1	429.9	259.5	233.0	196.4
AT2G39518	CASPL4D2	1,391.8	757.2	608.3	495.7	441.6	322.5
AT2G41905		37.8	7.5	1.9	15.8	16.5	18.6
AT3G02040	GDPD1, SRG3	94.3	55.7	5.1	41.2	37.2	45.6
AT3G11080	RLP35	12.5	2.6	0	5.0	5.0	5.9
AT3G48630		10.8	0.6	0	6.0	9.5	4.7
AT3G49780	PSK4	1,056.8	498.8	192.1	185.9	165.5	109.6
AT3G50470	HR3, MLA10	113.2	23.6	3.7	40.1	32.5	24.7
AT3G52400	SYP122	565.8	404.7	209.1	127.6	140.3	152.2
AT3G57380		12.5	7.5	0	1.8	1.2	0.8
AT3G61390	PUB36	103.0	48.0	18.4	36.7	37.4	37.0
AT4G08780		17.7	1.5	0	5.8	14.2	11.6
AT4G11910	NYE2, SGR2	12.4	2.9	0	7.8	6.3	4.1
AT4G12480	EARLI1	2,553.4	1,141.6	1,240.8	852.6	731.3	681.1
AT4G12490	AZI3	3,325.1	1,944.6	1,311.6	1,553.7	1,357.8	1,192.1
AT4G14630	GLP9	622.3	253.3	156.4	147.8	160.4	132.9
AT4G15270		7.0	0.5	0	2.6	1.8	2.4
AT4G15610	CASPL1D1	1,068.2	587.3	487.4	319.3	283.4	208.1
AT4G31800	WRKY18	547.1	383.0	264.9	245.4	255.7	202.3
AT4G34380		9.8	0.5	0	1.3	5.0	2.8
AT5G08380	AGAL1	43.3	20.0	3.7	33.3	30.7	36.4
AT5G13190	GILP	792.8	653.9	377.5	180.4	192.6	193.6
AT5G18470		83.0	76.5	11.2	66.3	94.2	71.0
AT5G20230	SAG14	2,035.2	1,195.4	817.8	358.6	643.3	524.0
AT5G24530	DMR6	699.2	471.9	322.6	357.2	327.1	278.3
AT5G26920	CBP60G	485.7	259.5	196.3	129.9	172.9	136.3
AT5G42300	UBL5	1,178.7	940.2	775.1	454.0	478.4	441.3
AT5G50200	NRT3.1, WR3	135.0	113.5	30.9	67.6	68.3	71.3
AT5G54140	ILL1	15.2	2.5	0	7.2	10.7	9.3
AT5G55470	NHX2	3.4	0.3	0	3.2	3.1	4.0
AT5G55560		35.9	12.7	1.9	14.7	8.7	11.0
AT5G56970	CKX3	13.1	1.8	0	3.1	8.2	5.1
AT5G57010		15.7	0.6	0	4.6	9.2	3.1
AT5G64120	PRX71	2,630.0	807.5	812.9	1,244.2	940.8	678.3

Arabidopsis genome initiative number.

Expression levels from 3 biological replicates are represented as the mean value of TPM of total reads aligned to Arabidopsis genome. “0” indicates no sequence read aligned.

**Figure 5.**
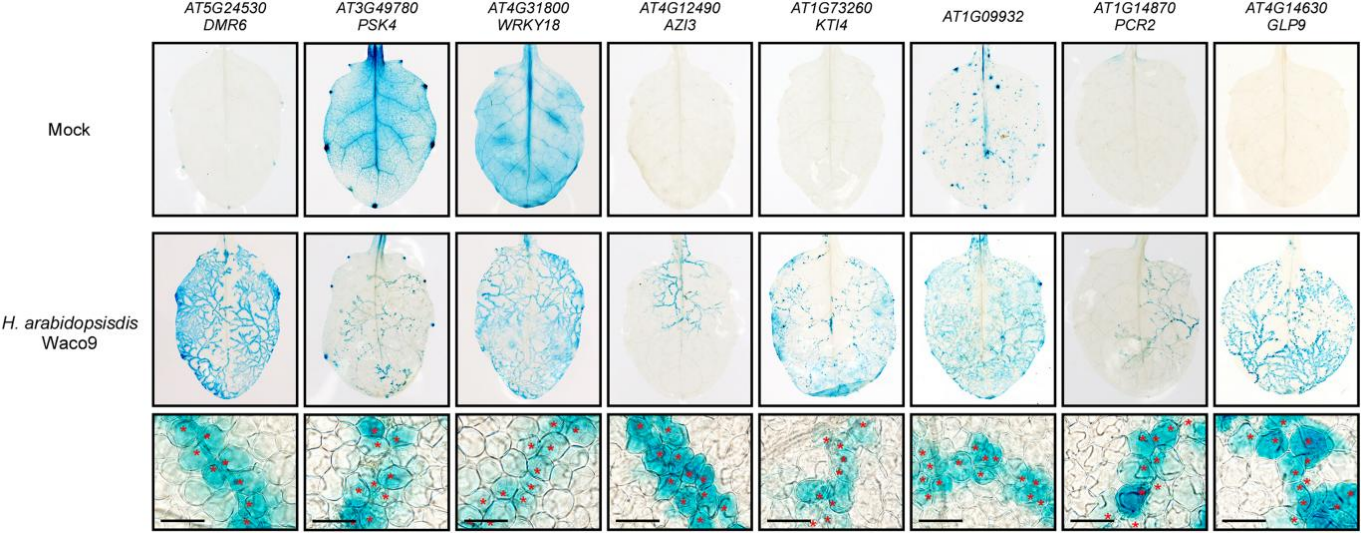
Cellular expression patterns of *DMR6*-coexpressed genes. GUS staining of 3-wk-old Arabidopsis leaves containing the indicated gene promoter fused to a *GUS* reporter gene 5 d after inoculating leaves with *H. arabidopsidis* Waco9 and water as a control (Mock). A GUS staining solution containing one-fifth the amount of substrate was used to monitor expression in the infected leaves due to high promoter activity in response to *H. arabidopsidis* infection. The images in the lower panel are magnifications of the middle images. Red asterisks indicate locations where *H. arabidopsidis* haustoria formed in leaf mesophyll cells. Scale bars = 40 *μ*m.

In the *DMR6*-coexpressed gene list (Table 1), we found *PHYTOSULFOKINE 4 PRECURSOR* (*PSK4*; *AT3G49780*) and *WRKY18* (*AT4G31800*), genes known to function as negative regulators of plant immunity. Arabidopsis transformants containing the *PSK4* or *WRKY18* promoter controlling the *GUS* reporter gene were generated and inoculated with *H. arabidopsidis* to confirm that *PSK4* and *WRKY18* are expressed in haustoriated cells. In both transformants, GUS staining was restricted to haustoriated cells as observed for *H. arabidopsidis*–infected *pDMR6*::*GUS* lines (Fig. 5). This result indicated that *PSK4* and *WRKY18* are expressed predominantly in the cells haustoriated with *H. arabidopsidis*. These data also suggest that genes involved in plant immunity can be identified using our TRAP system. Next, we randomly chose the following 5 genes from among the *DMR6*-coexpressed candidate genes (Table 1) for promoter-fused GUS analysis: *AZELAIC ACID INDUCED 3* (*AZI3*; *AT4G12490*), *KUNITZ TRYPSIN INHIBITOR 4* (*KTI4*; *AT1G73260*), *AT1G09932* (annotated to encode a phosphoglycerate mutase family protein), *PLANT CADMIUM RESISTANCE 2* (*PCR2*; *AT1G14870*), and *GERMIN-LIKE PROTEIN 9* (*GLP9*; *AT4G14630*). As expected, GUS staining was observed specifically in *H. arabidopsidis*–haustoriated cells in all transformants tested and in the *pDMR6*::*GUS* control (Fig. 5), indicating that these 5 genes are also coexpressed with *DMR6*.

### Identifying host genes whose overexpression confers resistance to downy mildew

**Figure 6.**
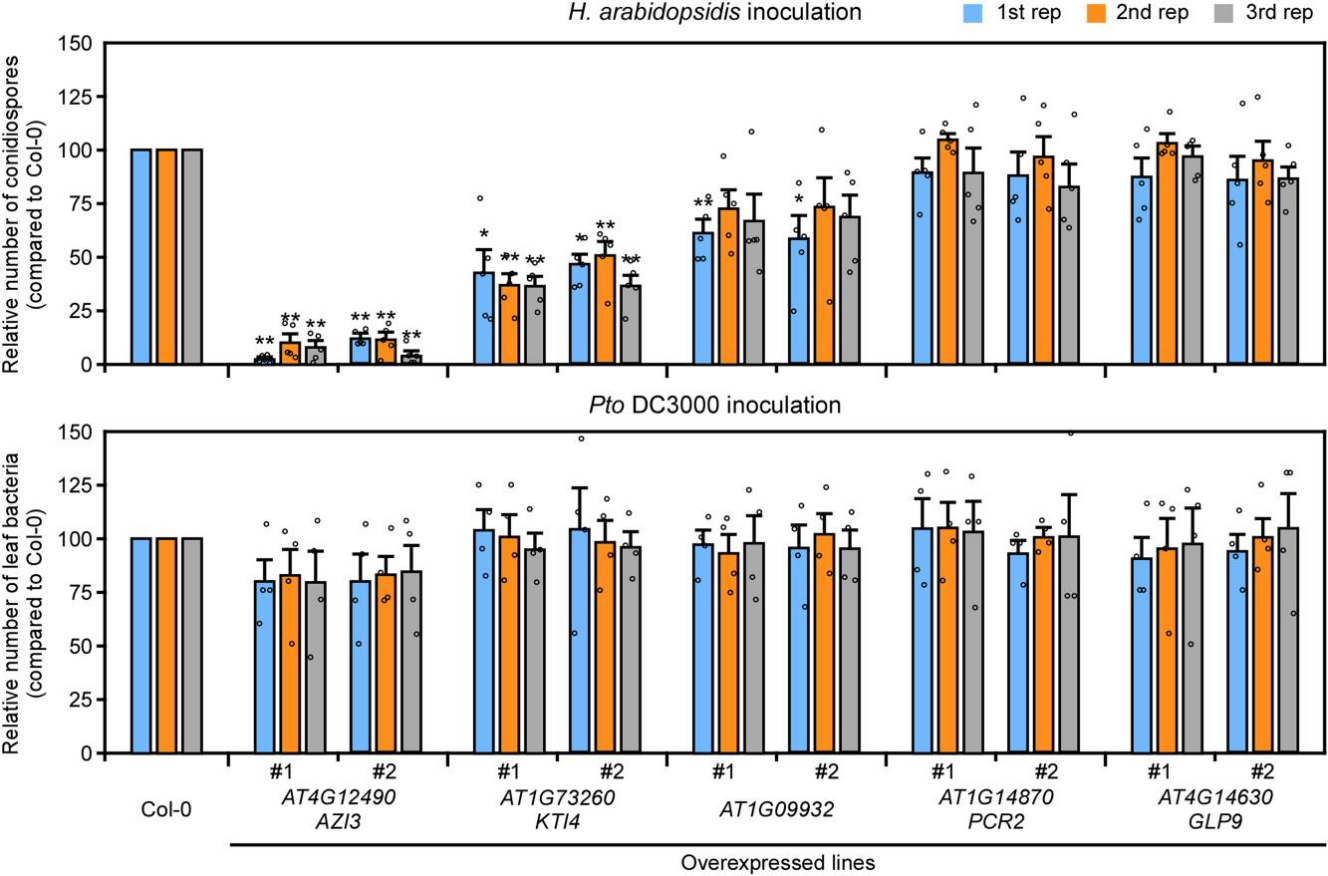
Disease resistance phenotypes of transgenic plants expressing *DMR6*-coexpressed genes. *Hyaloperonospora arabidopsidis* (upper panel) and *P. syringae* pv. *tomato* (*Pto*) DC3000 (lower panel) growth on 2 independent transgenic lines expressing the indicated genes. Data are shown relative to the Arabidopsis Col-0 WT value of 100. Data are means ± Ses from 5 and 4 biological replicates for *H. arabidopsidis* and *Pto* DC3000 growth, respectively, and represent 3 independent results. Data were analyzed by using Student's *t*-test: *P < 0.05; **P < 0.01 vs Col-0 WT plants.

To assess whether these 5 genes are involved in the Arabidopsis–*H. arabidopsidis* interaction, we created Arabidopsis transformants overexpressing each gene. Two independent lines for each gene were selected. All individuals were morphologically similar to Col-0 wild-type (WT) plants (Supplemental Fig. S5). At 5 d after inoculation with *H. arabidopsidis*, resistance levels of the transformants were assessed by counting the number of conidiospores formed on the plants and comparing them with Col-0 WT (Fig. 6). The most significantly resistant phenotypes were observed in *AZI3*-overexpressing lines that reproducibly had fewer than 15% of the conidiospores formed on Col-0 WT. The other resistant lines were *KTI4* overexpressors that had fewer than one-half of the conidiospores formed on Col-0 WT. Plants overexpressing *AT1G09932* appeared to have slightly increased resistance to *H. arabidopsidis*. In contrast, *PCR2*-overexpressing and *GLP9*-overexpressing lines showed no difference in resistance compared with Col-0 WT. Notably, none of the tested transformants differed from Col-0 WT in their resistance to the bacterial pathogen *Pseudomonas syringae* pv. *tomato* (*Pto*) DC3000 (Fig. 6), suggesting that at least the *AZI3*- and *KTI4*-overexpressing lines are specifically resistant to *H. arabidopsidis*. To investigate the effect of *azi3* loss on disease resistance, we searched for the available T-DNA mutants but did not find any insertions in *AZI3*; however, we did find a line with T-DNA inserted in the promoter region of *KTI4* (SALK_131716C, referred to as *kti4.1*), leading to reduced *KTI4* expression (Arnaiz et al. 2018). No substantial differences in disease resistance to *H. arabidopsidis* were observed for *kti4.1* compared with Col-0 WT (Supplemental Fig. S6).

## Discussion

RNA profiling is a powerful method for determining the molecular basis of host–pathogen interactions, but analyses using whole tissues lead to responses from a variety of cell types, including infected and noninfected cells. Here, we present an infected cell-specific RNA profiling strategy during the Arabidopsis–downy mildew interaction by employing a TRAP system using the E9-Im9 pair. Our study found genes that are specifically expressed in cells haustoriated by *H. arabidopsidis*. For example, this method detected *PSK4* and *WRKY18* that are predominantly expressed in haustoriated cells. Furthermore, overexpression of *AZI3* or *KTI4*, 2 genes found to be specifically expressed in haustoriated cells, conferred resistance to *H. arabidopsidis* but not to *Pto* DC3000.

Recently, a conceptually similar methodology using split GFPs was reported (Dinkeloo et al. 2022). Like ours, their method employed the *DMR6* promoter to drive the expression of a GFP fragment with a purification tag and another GFP fragment with a ribosome binding site, enabling the capture of polysomes from infected cells. Unfortunately, the report did not provide a list of genes detected by this method, making it impossible to compare it with our data set. One notable strategic difference is that we also used the *PR1* promoter, which is active in neighboring cells but not in haustoriated cells (Caillaud et al. 2013), to remove genes expressed in both cell types. This strategy provided an essential step as 2,953 out of 4,524 genes (65%) that *pDMR6*::*E9-HF* captured were also found by *pPR1*::*E9-HF* (Fig. 4B). Furthermore, 54 out of 1,571 (3.4%) genes were selected as induced at 5 dpi with *H. arabidopsidis* to eliminate genes expressed in haustoriated cells but not responsive to the pathogen (Fig. 4B). Finally, histochemical GUS analysis confirmed that at least 7 genes were specifically expressed in the haustoriated cells (Fig. 5). These results strongly support that our RNA profiling of the cells of interest was successful.

In this study, we focused on host genes whose expression is induced in haustoriated cells through bioinformatic filtering. The current data set can be filtered in different ways to obtain different outputs. For example, out of the 1,016 *PR1*-coexpressed candidate genes (pPR1-specific UP) found by the comparison of upregulated genes between *pDMR6::E9-HF* and *pPR1::E9-HF* transformants in the RNAs_IP samples (Fig. 4B and Supplemental Table S3), 48 genes were among those significantly upregulated at 5 dpi with *H. arabidopsidis* reported by Asai et al. (2014) (Supplemental Fig. S7). These could be strong candidates for *PR1*-coexpressed genes during interaction with *H. arabidopsidis*. Similarly, out of 2,953 genes that both *pDMR6*::*E9-HF* and *pPR1*::*E9-HF* captured, 138 genes were found as induced during infection with *H. arabidopsidis* (Supplemental Fig. S7), which would be genes expressed in both *DMR6* and *PR1*-expressing cells. Interestingly, 635 out of 875 genes (72.6%) upregulated during *H. arabidopsidis* infection reported previously (Asai et al. 2014) were not included in the current TRAP RNA-seq data (Supplemental Fig. S7). These 635 genes might be expressed in cells different from those in which *DMR6* and *PR1* are predominantly expressed when infected with *H. arabidopsidis*. Alternatively, this TRAP analysis might have missed some genes coexpressed with *DMR6* or *PR1* due to limitations of the TRAP method that concentrate but might not recover all of the ribosome-associated mRNAs derived from cells of interest. When comparing transcriptome data with TRAP RNA-seq data, it is also important to note that TRAP analysis determines only the abundance of ribosome-associated mRNAs, i.e. translated mRNAs, but not the abundance of transcripts.

Among the 7 genes found to be expressed in the haustoriated cells (Fig. 5), we found *PSK4* and *WRKY18* that are known to be involved in the modulation of plant immunity. Overexpression of *PSK4* and application of its active 5-amino-acid bisulfated phytosulfokine (PSK) peptide inhibit pattern-triggered immunity (PTI) responses and increase the susceptibility to *Pto* DC3000 (Igarashi et al. 2012; Mosher et al. 2013). Similarly, *WRKY18* is redundant with *WRKY40* and negatively regulates the expression of PTI-responsive genes and resistance toward *Pto* DC3000 and the powdery mildew fungus *Golovinomyces orontii* (Xu et al. 2006; Pandey et al. 2010; Birkenbihl et al. 2017). As *PSK4* and *WRKY18* are specifically induced in haustoriated cells, these genes can be considered as *S* genes that help pathogen infection, similar to *DMR6*. A previous chromatin immunoprecipitation sequencing (ChIP-seq) analysis reported 1,290 genes as WRKY18 target genes during PTI (Birkenbihl et al. 2017). In our experiments, 9 out of the 54 genes (17%), including *DMR6* and the 53 *DMR6*-coexpressed genes, were identified (Table 1) as targets of WRKY18 (Supplemental Table S4). Thus, WRKY18 may play a key role as a transcriptional hub for the *S* genes network. Since many *H. arabidopsidis* effectors are known to localize into plant cell nuclei when expressed in planta (Caillaud et al. 2012), targeting such hubs can be a suitable strategy for establishing infections as a biotroph.

In this study, we also identified *AZI3* as a transcriptionally induced gene in haustoriated cells whose overexpression conferred resistance to *H. arabidopsidis*. *AZI3* (*AT4G12490*) is a close paralog of the lipid transfer protein genes *AZI1* (*AT4G12470*) and *AZI4* (*AT4G12500*). These 3 genes have another paralog, *EARLY ARABIDOPSIS ALUMINUM INDUCED 1* (*EARLI1*; *AT4G12480*); all 4 genes are tandemly located on Chromosome 4 in Arabidopsis (Cecchini et al. 2015), and all 4 genes are induced upon *H. arabidopsidis* infection (Asai et al. 2014). In particular, *EARLI1* is included among the 53 *DMR6*-coexpressed genes (Table 1), whereas *AZI1* and *AZI4* are not included but appear to be coexpressed with *DMR6* (Supplemental Fig. S8). Among the 4 paralogs, *AZI1* and *EARLI1* are reportedly key factors in establishing systemic acquired resistance (SAR) by affecting the lipid-derivative azelaic acid (AZA) mobilization from local tissues to distal sites (Jung et al. 2009; Cecchini et al. 2015). AZI1, AZI3, and EARLI1 all localize in the endoplasmic reticulum (ER)/plasmodesmata, chloroplast outer envelopes, and membrane-contact sites between these organelles (Cecchini et al. 2015). Since AZA is produced in chloroplasts (Zoeller et al. 2012), AZI1 and its paralogs are thought to form part of the complexes contacting both chloroplasts and ER membranes, potentially allowing the nonvesicular transport of AZA to distal tissues (Cecchini et al. 2015). In this scenario, Arabidopsis may induce SAR signaling to counter secondary infection by expressing *AZI1* and its paralogs in the *H. arabidopsidis*–infected cells. Consistent with this hypothesis, *AZI3*-overexpressing lines exhibited enhanced resistance to *H. arabidopsidis* (Fig. 6). Interestingly, the *AZI3*-based enhanced resistance is *H. arabidopsidis* specific as *AZI3* overexpressors showed no difference in bacterial growth on local leaves after inoculation with *Pto* DC3000, a finding consistent with the results in *AZI1* overexpressing lines reported by Wang et al. (2016). The effect of *azi3* loss on disease resistance was not investigated, since the corresponding T-DNA mutants were unavailable. As SAR is reduced in the *azi1* and *earli1* mutants (Jung et al. 2009; Cecchini et al. 2015), it should be instructive to determine the effect of the quadruple mutation of *AZI1* and its paralogs on plant immunity.

*KTI4*, a gene that encodes a functional Kunitz trypsin inhibitor (Li et al. 2008), is another gene identified in our study. The observation that *KTI4* overexpressors exhibit higher resistance to *H. arabidopsidis* (Fig. 6) is markedly different from the findings of a previous study that reported overexpression of *KTI4* leads to higher susceptibility to the bacterial necrotroph *Pectobacterium carotovorum* (formerly *Erwinia carotovora*; Li et al. 2008). The opposite resistance phenotypes against these pathogens might be due to a difference in lifestyle between biotrophs and necrotrophs. As SA signaling functions oppositely in biotrophs and necrotrophs (Hou and Tsuda 2022) and *KTI4* is induced by SA (Li et al. 2008), *KTI4* may be involved in SA signaling. The expression of *DMR6* inactivates SA in *H. arabidopsidis*–haustoriated cells and may suppress plant immunity activated by *KTI4*, resulting in infection. *KTI4*-overexpressing lines did not show increased resistance to *Pto* DC3000 (Fig. 6) or have any effect on plant growth (Supplemental Fig. S5), suggesting that *KTI4*-mediated immunity is not constantly activated. The *kti4.1* mutant showed no difference in resistance to *H. arabidopsidis* compared with Col-0 WT (Supplemental Fig. S6), possibly because of redundancy, as there are 6 pulative paralogs of *KTI4* in Arabidopsis (Arnaiz et al. 2018). In fact, the closest putative paralog *KTI5* (*At1G17860*) seems to be coexpressed with *DMR6* (Supplemental Fig. S9), although the putative paralog was not included in the list of 53 *DMR6*-coexpressed genes (Table 1). Further analysis is needed to determine how *KTI4* may be involved in resistance to *H. arabidopsidis*.

Our TRAP system revealed host genes induced in the *H. arabidopsidis*–infected cells that function either in susceptibility or resistance. We hypothesize that different mechanisms induce the expression of these genes. For instance, susceptibility-related genes may be induced by *H. arabidopsidis*, perhaps by using its effectors. In contrast, Arabidopsis may actively induce resistance-related genes by recognizing pathogen-derived molecules. Further genetic analysis is needed to dissect the signaling pathways. In addition, we expect that this E9-Im9-based TRAP system could be applicable to several other stimulus-specific contexts and other plant–pathogen interactions using relevant specific promoters.

## Materials and methods

### Plant materials and growth conditions

Arabidopsis (*A. thaliana*) plants were grown at 22°C with a 10 h photoperiod and a 14 h dark period in environmentally controlled growth cabinets. *Nicotiana benthamiana* plants were grown at 25°C with a 16 h photoperiod and an 8 h dark period in environmentally controlled growth cabinets.

### Pathogen assays

Inoculation with the *H. arabidopsidis* Waco9 isolate was conducted as described by Asai et al. (2015). Briefly, 3-wk-old Arabidopsis plants were spray inoculated to saturation with a spore suspension of 1 × 10^4^ conidiospores mL^−1^. Plants were covered with a transparent lid to maintain high humidity (90% to 100%) conditions in a growth cabinet at 16°C with a 10 h photoperiod for 5 d. Five replicates of 3 plants for each Arabidopsis line were used in the bioassays (Fig. 6). Conidiospores were harvested in 1 mL of water. After vortexing, the number of released conidiospores was determined using a hemocytometer. *Pseudomonas syringae* pv. *tomato* DC3000 was grown on LB media containing 100 *μ*g mL^−1^ rifampicin at 28°C. Five- to 6-wk-old soil-grown plants were syringe infiltrated with a bacterial suspension of 5 × 10^5^ cfu mL^−1^ in 10 mM MgCl_2_. Bacterial growth in plants was monitored at 3 d post inoculation.

### Plasmid construction

For the construction of the TRAP plasmids, the ORF of *RPL18* together with the 3′ UTR and the terminator was amplified from Col-0 gDNA for Golden Gate assembly (Engler et al. 2008; Engler et al. 2014) into the pICH47751 vector with the *35S* promoter and *Im9* (with GS spacer) as an N-terminal fusion tag. The 2,486 bp *DMR6*, 2,378 bp *PR1*, and 1,450 bp *Act2* promoters were amplified from Col-0 gDNA for Golden Gate assembly (Engler et al. 2008; Engler et al. 2014) into the pICH47761 vector with *E9*, *HF* as a C-terminal fusion tag and the octopine synthase (*OCS*) terminator. For the final Golden Gate assembly, *p35S*::*Im9-RPL18* (pICH47751) was combined with *pDMR6/pPR1/pAct2*::*E9-HF* (pICH47761), the herbicide BASTA-resistance gene (*BAR*; pICH47732) and *FastRed* (pICH47742) into the Level 2 Golden Gate vector pAGM4723.

For the transient expression studies, the ORF of *RPL18* was amplified from Col-0 cDNA for Golden Gate assembly (Engler et al. 2008; Engler et al. 2014) into the binary vector pICH86988 with *Im9* or *YFP* as an N-terminal fusion tag. *E9* fused to *GFP* as a C-terminal fusion tag was also cloned into the pICH86988 vector.

For GUS reporter constructs, the promoter sequence plus 27 or 30 bp upstream from the start codon of *PSK4* (1,827 bp), *WRKY18* (2,030 bp), *AT1G09932* (1,062 bp), *PCR2* (2,030 bp), *KTI4* (993 bp), *AZI3* (2,030 bp), and *GLP9* (2,030 bp) was amplified from Col-0 gDNA for Golden Gate assembly (Engler et al. 2008, 2014) into the binary vector pICSL86955 with the *GUS* reporter gene and *OCS* terminator.

For overexpressing constructs, the ORFs of *AT1G09932*, *PCR2*, *KTI4*, *AZI3*, and *GLP9* were amplified from Col-0 gDNA for Golden Gate assembly (Engler et al. 2008, 2014) into the binary vector pICSL86977 containing *p35S* with a C-terminal *HF* fusion tag.

### Transient gene expression and plant transformation

For transient gene expression analysis, *Agrobacterium tumefaciens* strain AGL1 was used to deliver the respective transgenes to *N. benthamiana* leaves using methods previously described (Asai et al. 2008). All bacterial suspensions carrying individual constructs were adjusted to an OD_600_ = 0.5 in the final mix for infiltration, except for the coexpression of *35S*::*E9-GFP* with *35S*::*Im9-RPL18* in which bacterial suspensions were adjusted to OD_600_ = 0.25 for *35S*::*E9-GFP* and OD_600_ = 0.5 for *35S*::*Im9-RPL18* due to low expression levels of Im9-RPL18. We hypothesize that the turnover of RPL18 occurs more rapidly than for E9-GFP.

For plant transformation, Arabidopsis Col-0 plants were transformed using the dipping method (Clough and Bent 1998). Briefly, flowering Arabidopsis plants were dipped into a solution containing *A. tumefaciens* carrying a plasmid of interest, and the seeds were harvested to select the T1 transformants on selective MS media. T1 plants were checked for expression of the construct-of-interest by immunoblot analysis. T2 seeds were sown on selective MS media, and the proportion of resistant versus susceptible plants was measured to identify lines with single T-DNA insertions. Transformed plants were transferred to soil, and the seeds were collected. Two independent T3 homozygous lines were analyzed.

### Confocal microscopy

For in planta subcellular localization analysis in *N. benthamiana*, cut leaf patches were mounted in water and analyzed using a Leica TCS SP8 X confocal microscope (Leica Microsystems) with an HC PL APO CS2 40×/1.10 water-corrected immersion objective at digital zoom 6.5. The excitation/emission wavelengths were 488/503 to 546 nm for E9-GFP and 513/518 to 569 nm for YFP-RPL18 with white-light laser intensity 85% and gain 100.

### Protein extraction and immunoblotting

Leaves were ground to a fine powder in liquid nitrogen and thawed in extraction buffer (50 mM Tris-HCl, pH 7.5, 150 mM NaCl, 10% [v/v] glycerol, 10 mM DTT, 10 mM EDTA, 1 mM NaF, 1 mM Na_2_MoO_4_·2H_2_O, 1% [v/v] IGEPAL CA-630 from Sigma-Aldrich, and 1% [v/v] protease inhibitor cocktail from Sigma-Aldrich). Samples were cleared by centrifugation at 16,000 × *g* for 15 min at 4°C, and the supernatant was collected and subjected to SDS-PAGE. Proteins were then electroblotted onto a PVDF membrane using a semidry blotter (Trans-Blot Turbo Transfer System; Bio-Rad). Membranes were blocked overnight at 4°C in TBS-T (50 mM Tris-HCl, pH 7.5, 150 mM NaCl, and 0.05% [v/v] Tween 20) with 5% (w/v) skim milk. Membranes were then incubated with horseradish peroxidase-conjugated anti-FLAG antibody (1:20,000; A8592; Sigma-Aldrich) diluted with TBS-T with 5% (w/v) skim milk at room temperature for 1 h. After washing with TBS-T, bound antibodies were visualized using SuperSignal West Femto Maximum Sensitivity Substrate (Thermo Fisher Scientific). Bands were imaged using an image analyzer (ImageQuant LAS 4000 imager; GE Healthcare).

### Translating ribosome affinity purification

TRAP was performed according to the method of Mustroph et al. (2009a) with the following modifications: eighty-one 3-wk-old plants 5 d after inoculation with *H. arabidopsidis* were ground in liquid nitrogen and 8 mL of polysome extraction buffer (PEB) was added. The resulting extract was clarified twice by centrifugation at 16,000 × *g* for 15 min at 4°C, with a Miracloth filtration step between centrifugations. From a portion of the clarified extract, RNA was extracted and referred to as RNAs_Total. The remainder of the extract was mixed with 150 *μ*L washed α-FLAG agarose beads (A2220; Sigma) and adjusted to 5 mL with PEB. The extract was incubated with the beads for 2 h with gentle rocking at 4°C. The beads were washed as follows: 1 wash with 6 mL PEB and 4 washes with 6 mL wash buffer. The washed beads were resuspended in 300 *μ*L wash buffer containing 300 ng *μ*L^−1^ of 3xFLAG peptide (F4799; Sigma) and 20 U mL^−1^ RNAsin (Promega) and incubated for 30 min with gentle rocking at 4°C. RNA was extracted from the supernatant liquid collected after centrifugation and is referred to as RNAs_IP.

### RNA extraction, cDNA synthesis, and RT-qPCR

Total RNAs were extracted using RNeasy Plant Mini Kit (Qiagen), according to the manufacturer's procedure. Total RNAs (1 *μ*g) were used for generating cDNAs in a 20 *μ*L reaction according to the Invitrogen Superscript III Reverse Transcriptase protocol. The obtained cDNAs were diluted 5 times, and 1 *μ*L was used for a 10 *μ*L RT-qPCR reaction. RT-qPCR was performed in a 10 *μ*L final volume using 5 *μ*L SYBR Green Mix (Toyobo), 1 *μ*L diluted cDNAs, and primers. RT-qPCR was run on Mx3000P qPCR System (Agilent) using the following program: (i) 95°C, 3 min; (ii) (95°C, 30 s, then 60°C, 30 s, then 72°C, 30 s) × 45, (iii) 95°C, 1 min followed by a temperature gradient from 55°C to 95°C. The relative expression values were determined using the comparative cycle threshold method (2^−ΔΔCt^). *ELONGATION FACTOR 1 ALPHA* (*EF-1α*) was used as the reference gene. Primers used for RT-qPCR are listed in Supplemental Table S5.

### RNA sequencing

The library prepared for RNA sequencing was constructed as described previously (Rallapalli et al. 2014). Purified double-stranded cDNAs were subjected to Covaris shearing (parameters: intensity, 5; duty cycle, 20%; cycles/burst, 200; duration, 60 s). The libraries were sequenced on an Illumina NextSeq 500 DNA sequencer. Sequence data have been deposited in NCBI's Gene Expression Omnibus (GEO) and are accessible through GEO Series accession number GSE220449. The Illumina sequence library was quality-filtered using FASTX Toolkit version 0.0.13.2 (Hannonlab) with parameters -q20 and -p50. Reads containing “N” were discarded. Quality-filtered libraries were aligned on the Arabidopsis Col-0 genome with the Araport11 annotation using the default settings of CLC Genomic Workbench 20. Transcription levels for each transcript were calculated as transcripts per million (TPM). Differential expression was analyzed using the R statistical language version 4.1.1 with edgeR version 3.34.0 (Robinson et al. 2010), part of the Bioconductor package (Gentleman et al. 2004). The multidimensional scaling (MDS) plot was created using ggplot2 version 3.3.5. GO analysis of the 54 confident candidate *DMR6*-coexpressed genes shown in Table 1 was conducted by PANTHER (Mi et al. 2019) at The Arabidopsis Information Resource website (https://www.arabidopsis.org/tools/go_term_enrichment.jsp).

### GUS staining

GUS activity was assayed histochemically with 5-bromo-4-chloro-3-indolyl-β-D-glucuronic acid (1 or 0.2 mg mL^−1^) in a buffer containing 100 mM sodium phosphate pH 7.0, 0.5 mM potassium ferrocyanide, 0.5 mM potassium ferricyanide, 10 mM EDTA, and 0.1% (v/v) Triton. Arabidopsis leaves were vacuum-infiltrated with staining solution and then incubated overnight at 37°C in the dark. Samples were destained in absolute ethanol followed by incubation in a chloral hydrate solution. Stained leaves were observed using an Olympus BX51 microscope.

### Statistical analyses

A two-tailed unpaired Student's *t*-test was used for statistical analysis. Asterisks indicate significant differences between transformants and WT plants (*P < 0.05, **P < 0.01).

### Accession numbers

Sequence data from this article can be found in the GenBank/EMBL data libraries under accession numbers: AT5G24530 (DMR6), AT2G14610 (PR1), AT3G18780 (Act2), AT3G05590 (RPL18), P09883 (colicin E9), P13479 (Im9), AT3G49780 (PSK4), AT4G31800 (WRKY18), AT4G12490 (AZI3), AT1G73260 (KTI4), AT1G09932, AT1G14870 (PCR2), AT4G14630 (GLP9), AT4G12470 (AZI1), AT4G12480 (EARLI1), AT4G12500 (AZI4), AT1G73330 (KTI1), AT1G72290 (KTI2), AT1G73325 (KTI3), AT1G17860 (KTI5), AT3G04320 (KTI6), and AT3G04330 (KTI7).

## Supplementary Material

kiad326_Supplementary_Data

## Supplemental data

The following materials are available in the online version of this article.

**Supplemental Figure S1.** Schematic diagram of the traditional TRAP system.

**Supplemental Figure S2.** Expression levels of *DMR6* and *PR1* in samples derived from whole tissues during *H. arabidopsidis* infection.

**Supplemental Figure S3.** The MDS plot for RNA-seq data.

**Supplemental Figure S4.** Enriched GO terms of *DMR6*-coexpressed genes.

**Supplemental Figure S5.** Morphology of transgenic plants overexpressing *DMR6*-coexpressed genes.

**Supplemental Figure S6.** Disease resistance phenotypes of *kti4.1* to *H. arabidopsidis* inoculation.

**Supplemental Figure S7.** Overlapping status of the number of differentially expressed genes.

**Supplemental Figure S8.** Expression levels of *AZI1*, *EARLI1*, *AZI3*, and *AZI4* in the TRAP samples.

**Supplemental Figure S9.** Expression levels of *KTI4* and its putative paralogs in the TRAP samples.

**Supplemental Table S1.** The expression patterns of Arabidopsis genes in the TRAP samples after inoculation with *H. arabidopsidis* Waco9.

**Supplemental Table S2.** Genes with significantly different expression levels.

**Supplemental Table S3.** Lists of differentially expressed candidate genes.

**Supplemental Table S4.** List of genes overlapped between *DMR6*-coexpressed genes with WRKY18 target genes.

**Supplemental Table S5.** Primers used in this study.
