# Supplementary material for: Cell-specific RNA profiling reveals host genes expressed in Arabidopsis cells haustoriated by downy mildew
Source: Plant Physiol. 2023 Jun 12;193(1):259–70. doi: 10.1093/plphys/kiad326 (PMC10469357; doi:10.1093/plphys/kiad326)
Supplement: kiad326_Supplementary_Data [file kiad326_supplementary_data.zip › Supplemental Figures.pdf]

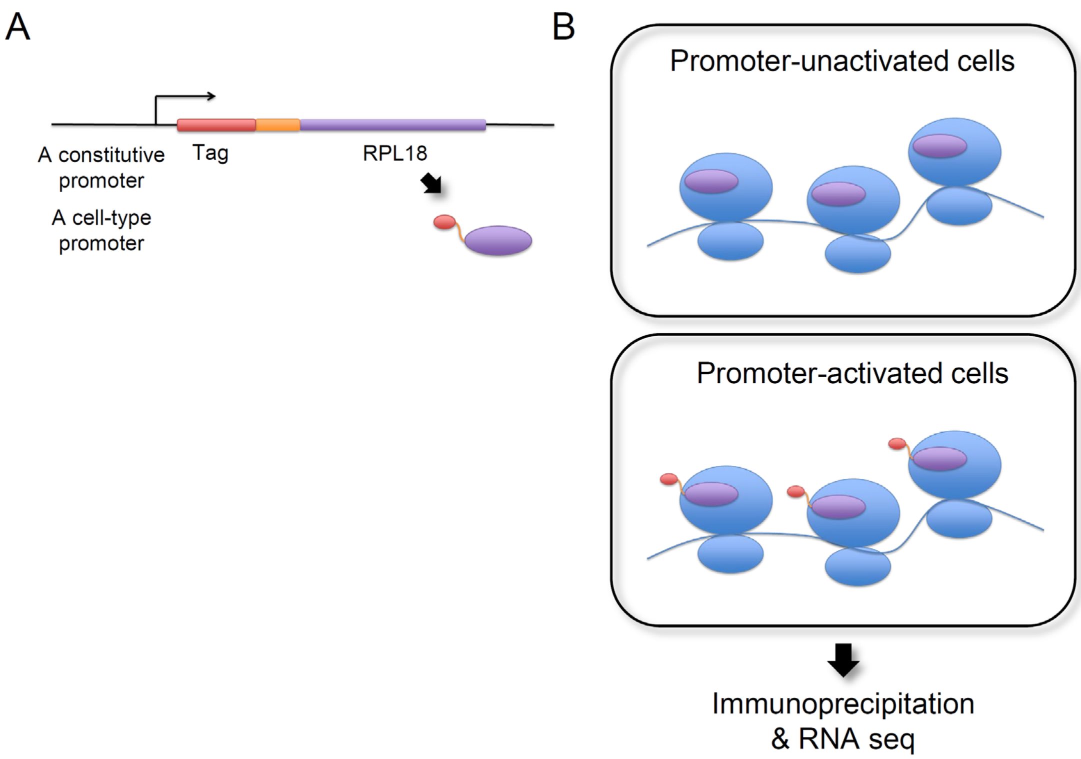

Supplemental Figure S1. Schematic diagram of the traditional translating ribosome affinity purification (TRAP) system. (**A**) A schematic representation of the chimeric construct; an epitope-tagged ribosomal protein L18 (RPL18) fused to a constitutive promoter or a promoter that is active in a specific cell type (cell-type promoter). (**B**) Schematic diagram of ribosomal complexes in cells where the promoters fused to an epitope-tagged RPL18 are unactivated or activated.

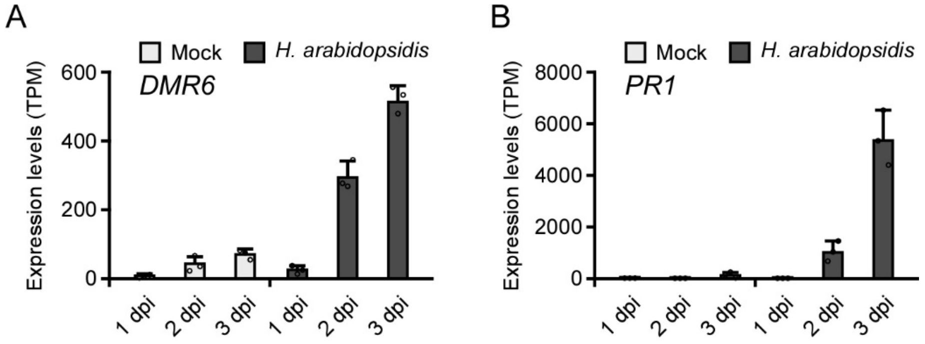

Supplemental Figure S2. Expression levels of *DMR6* and *PR1* in samples derived from whole tissues during *H. arabidopsidis* infection. Expression levels of *DMR6* (**A**) and *PR1* (**B**) at 1, 3, and 5 d post inoculation (dpi) with *H. arabidopsidis* Waco9 isolate or water as a control (Mock) are represented as TPM (tags per million) of total reads aligned to the Arabidopsis genome. Data are means ± SDs from three biological replicates. The data are derived from RNA seq data from Asai et al. 2014.

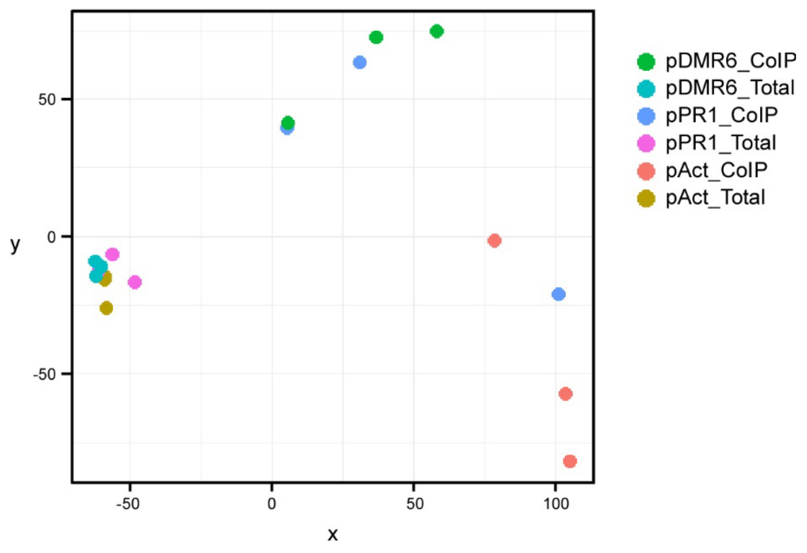

Supplemental Figure S3. The MDS plot for RNA-seq data. Each color represents the indicated sample with three independent biological replicates. The MDS plot was created using ggplot2.

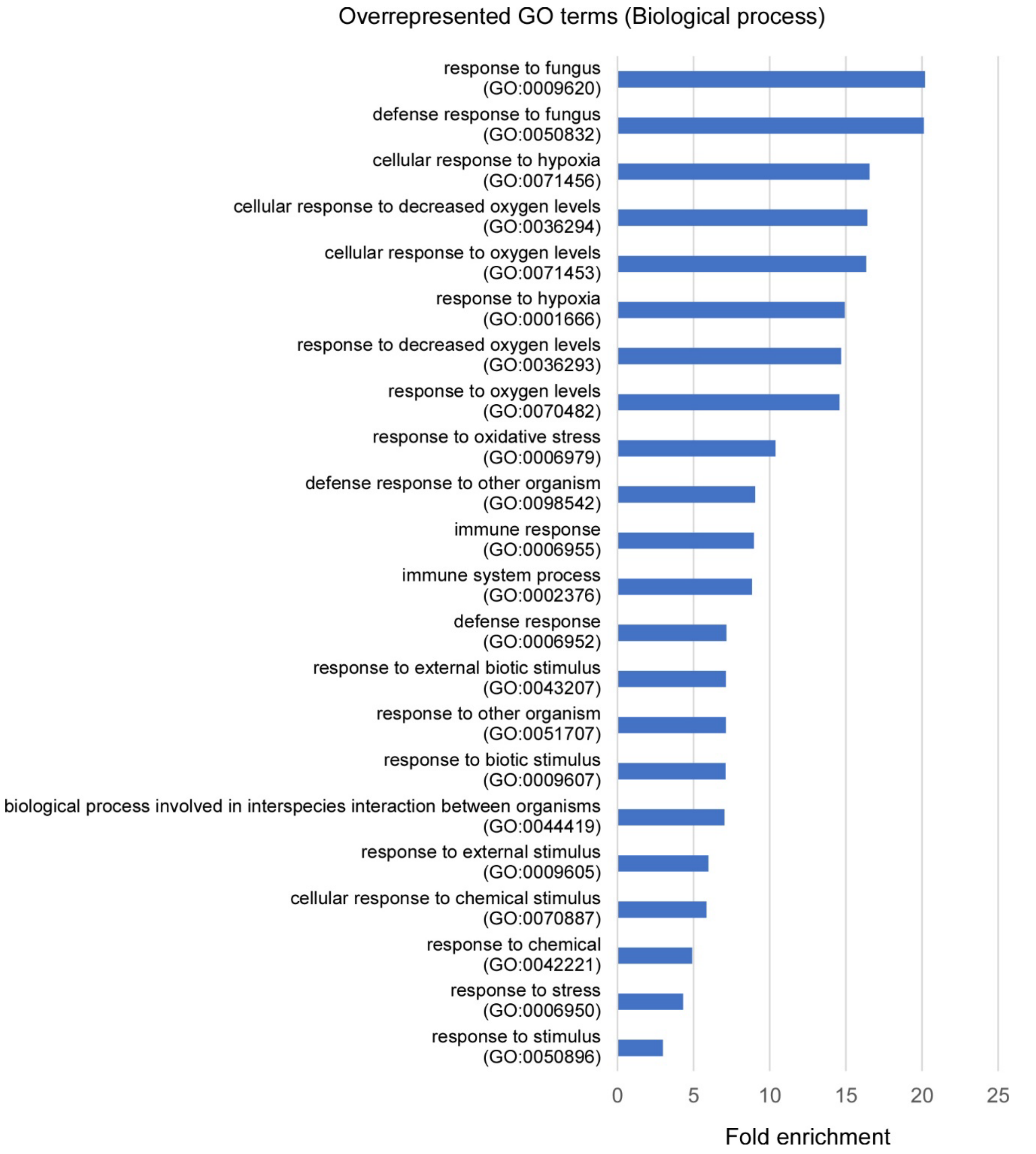

Supplemental Figure S4. Enriched gene ontology (GO) terms of *DMR6*-coexpressed genes. GO enrichment analysis of *DMR6* and 53 *DMR6*-coexpressed genes. Fold enrichment ($p < 0.05$) was determined by query gene number divided by the expected gene number for each GO term.

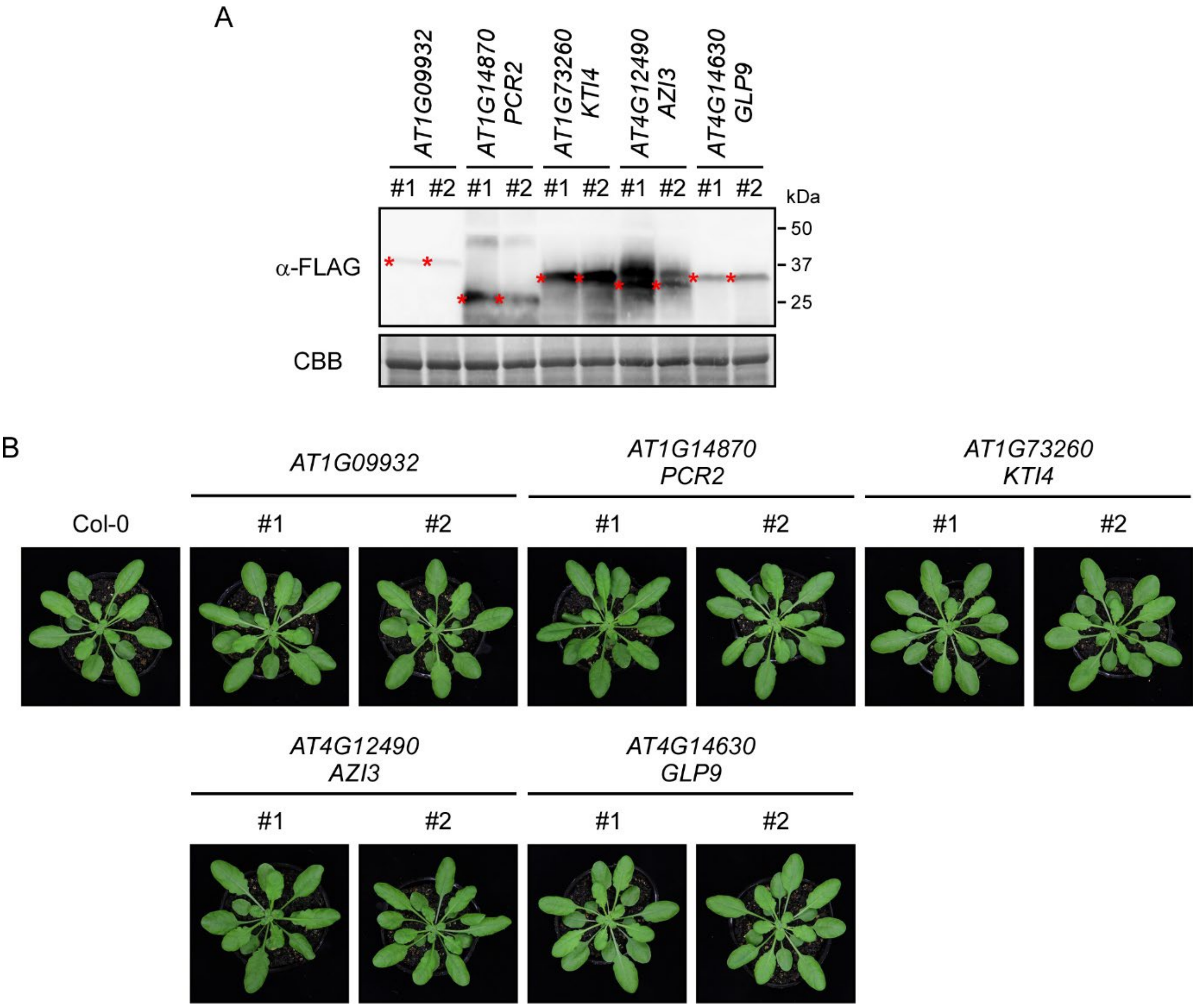

Supplemental Figure S5. Morphology of transgenic plants overexpressing *DMR6*-coexpressed genes. (**A**) Confirmation of protein accumulation in Arabidopsis Col-0 transgenic lines overexpressing *AT1G09932*, *PCR2*, *KTI4*, *AZI3*, and *GLP9*. Total proteins were prepared from 6-week-old plants. Immunoblotted proteins were treated with anti-FLAG (upper panel) antibodies. Asterisks indicate the detected transgene constructs. Protein loads were monitored by Coomassie Brilliant Blue (CBB) staining of the bands corresponding to ribulose-1,5-bisphosphate carboxylase (Rubisco) large subunit (lower panel). (**B**) Morphology of Arabidopsis Col-0 WT and transgenic lines photographed at 6-weeks.

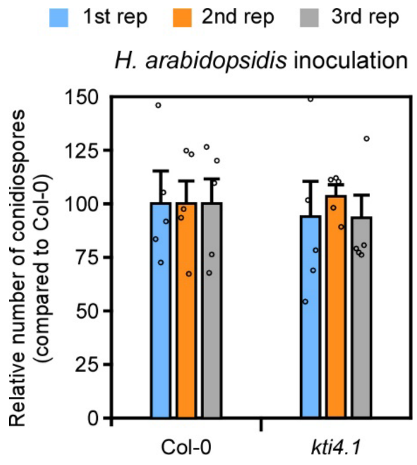

Supplemental Figure S6. Disease resistance phenotypes of *kti4.1* to *H. arabidopsidis* inoculation. *H. arabidopsidis* growth (conidiospore number) on the *kti4.1* mutant. Data are shown relative to the Arabidopsis Col-0 WT value of 100. Data are means ± SEs from five biological replicates and represent three independent results.

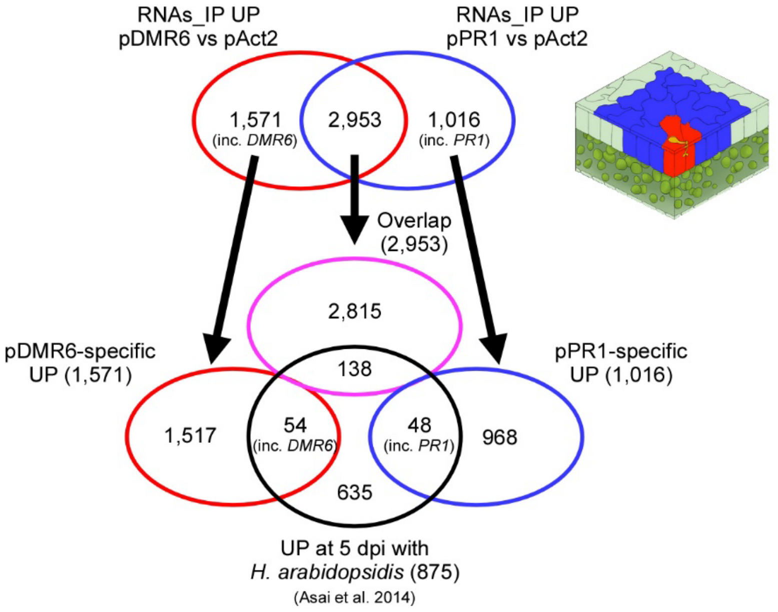

Supplemental Figure S7 Overlapping status of the number of differentially expressed genes. The comparison of upregulated genes between *pDMR6*::*E9-HF* and *pPR1*::*E9-HF* transformants in the RNAs_IP samples revealed 1,571, 1,016 and 2,953 genes as *DMR6*-coexpressed candidate genes (pDMR6-specific UP), *PR1*-coexpressed candidate genes (pPR1-specific UP), and candidate genes expressed in both *DMR6* and *PR1*-expressing cells (Overlap), respectively. Comparison of these genes with 875 genes significantly upregulated at 5 days post inoculation (dpi) with *H. arabidopsidis* reported by Asai et al. (2014) showed that the indicated number of genes overlapped. The diagram on the right indicates proposed expression sites: red-shaded cells, expression sites where *DMR6*-coexpressed genes are expressed; blue-shaded cells, expression sites where *PR1*-coexpressed genes are expressed.

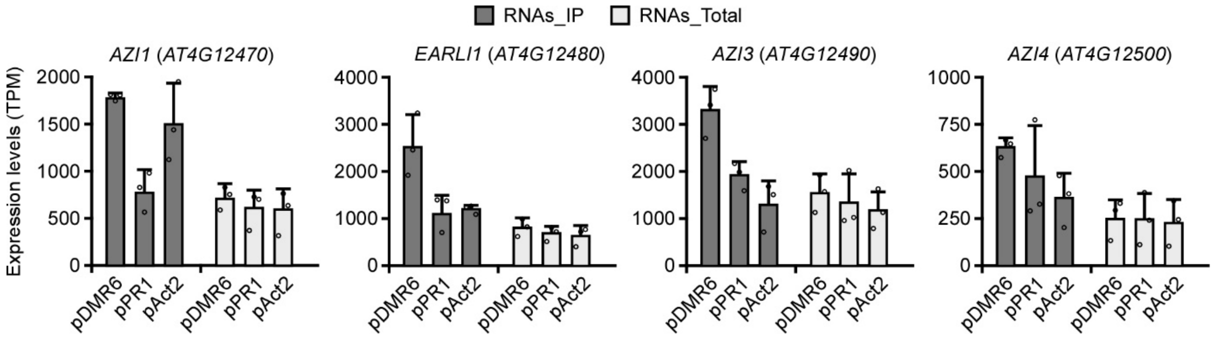

Supplemental Figure S8. Expression levels of *AZI1*, *EARLI1*, *AZI3,* and *AZI4* in the TRAP samples. Expression of *AZI1* (*AT4G12470*), *EARLI1* (*AT4G12480*), *AZI3* (*AT4G12490*) and *AZI4* (*AT4G12500*) in the Arabidopsis Col-0 transgenic lines containing *pDMR6*::*E9-HF* (pDMR6), *pPR1*::*E9-HF* (pPR1) or *pAct2*::*E9-HF* (pAct2) and *p35S*::*Im9-RLP18*. The expression level of *AZI1*, *EARLI1*, *AZI3,* and *AZI4* in the RNAs_Total and RNAs_IP samples are represented as TPM (transcripts per million) of total reads aligned to the Arabidopsis genome. Data are means ± SDs from three biological replicates.

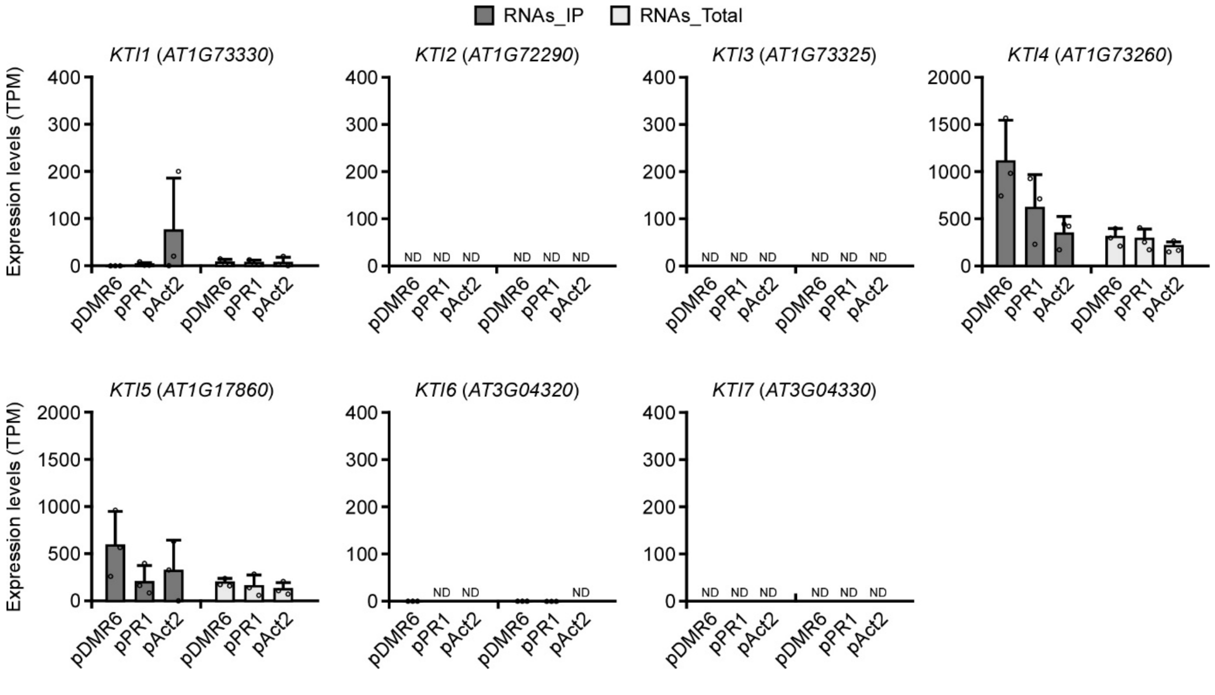

Supplemental Figure S9. Expression levels of *KTI4* and its putative paralogs in the TRAP samples. Expression of *KTI4* and its putative paralogs in the Arabidopsis Col-0 transgenic lines containing *pDMR6*::*E9-HF* (pDMR6), *pPR1*::*E9-HF* (pPR1) or *pAct2*::*E9-HF* (pAct2) and *p35S*::*Im9-RLP18*. The expression level of *KTI4* and its putative paralogs in RNAs_Total and RNAs_IP samples are represented as TPM (transcripts per million) of total reads aligned to the Arabidopsis genome. Data are means ± SDs from three biological replicates. ND, not detectable.
